# Successful Treatment of Multiple Large Intrarenal Stones in a 2-Year-Old Boy Using a Single-Use Flexible Ureteroscope and High-Power Laser Settings

**DOI:** 10.3390/pediatric16030068

**Published:** 2024-09-11

**Authors:** Vasileios Tatanis, Theodoros Spinos, Zoi Lamprinou, Elisavet Kanna, Francesk Mulita, Angelis Peteinaris, Orthodoxos Achilleos, Ioannis Skondras, Evangelos Liatsikos, Panagiotis Kallidonis

**Affiliations:** 1 Department of Urology, University of Patras Hospital, 26504 Patras, Greece; thspinos@otenet.gr (T.S.); peteinarisaggelis@gmail.com (A.P.); liatsikos@yahoo.com (E.L.); pkallidonis@yahoo.com (P.K.); 2 2nd Department of Pediatric Surgery, P & A Kyriakou Children’s Hospital, 11527 Athens, Greece; lamprinouzoe@gmail.com (Z.L.); elizakanna@gmail.com (E.K.); thoxisach@gmail.com (O.A.); skondras@yahoo.gr (I.S.); 3 Department of Surgery, University Hospital of Patras, 26504 Patras, Greece; med5507@ac.upatras.gr; 4 Department of Urology, Medical University of Vienna, 1090 Vienna, Austria

**Keywords:** stone disease, RIRS, high power, single use, flexible ureteroscopy

## Abstract

The standard treatment procedures for managing renal calculi in the pediatric population are similar to those in adults. The application of flexible ureteroscopy has contributed to the increased popularity of retrograde intrarenal surgery (RIRS) as an alternative therapeutic modality that can be successfully applied in children. One of the most significant innovations of the last decade is the introduction of single-use flexible ureteroscopes (fURSs). In this case report, we present the case of a 2-year-old boy with multiple large calculi in his right kidney, which were successfully removed after a single session of RIRS using a 7.5 F single-use fURS and high-power laser settings. The total operative and lithotripsy times were estimated at 90 and 75 min, respectively. No complications were recorded. The hemoglobin loss was calculated at 0.3 mg/dL, while the creatinine level was decreased by 0.1 mg/dL. The urethral catheter was removed on the first postoperative day, and the patient was discharged. The management of multiple or large kidney stones is very challenging in the pediatric population under the age of three years. Convenient preoperative planning and the appropriate use of available equipment may lead to excellent outcomes accompanied by a reduced risk for complications.

## 1. Introduction

Over the last two decades, the prevalence of pediatric urolithiasis has increased, and the most studied causative factors are either underlying anatomical and metabolic disorders or recurrent urinary tract infections [1]. The management and treatment of renal calculi in this age group remain controversial and are not yet well-established. The standard treatment procedures are similar to those in adults, and the application of flexible ureteroscopy has contributed to the increased popularity of retrograde intrarenal surgery (RIRS) as an alternative therapeutic modality that can be successfully applied in children [2]. Based on the EAU guidelines, RIRS constitutes the primary treatment option for pelvic stones of 10–20 mm in size, while it is an alternative option for pelvic stones of over 2 cm and lower calyceal stones [3]. Technological advancements and innovations in endoscopic equipment, along with the introduction and establishment of the holmium/YAG (Ho/YAG) laser, have rendered RIRS a safe and efficient approach for pediatric patients undergoing endoscopic lithotripsy [4]. Additionally, the integration of new technologies resulted in the amelioration of RIRS, promising better efficiency, even for large renal stones [5]. One of the most significant innovations of the last decade was the introduction and widespread use of single-use flexible ureteroscopes, which have recently gained increased popularity [6]. Single-use flexible ureteroscopes (fURSs) can overcome many of the drawbacks, which are associated with reusable flexible ureteroscopes, such as their increased cost and their limited lifespan and fragility [7]. Herein, we present a case of a 2-year-old boy with multiple large calculi (over 1 cm each) in different calyces in his right kidney, which was successfully removed after a single session of RIRS, using a 7.5 F single-use fURS and high-power (HP) laser settings.

## 2. Case Description

### 2.1. First Referral and Further Investigation

A 22-month-old boy with the diagnosis of renal stones in the right kidney was referred for further investigation and treatment due to recurrent urinary tract infections.

The medical history of this 22-month-old boy included five episodes of febrile urinary tract infections during the last year (positive urine cultures with Escherichia coli or Proteus mirabilis). An ultrasound was performed, which described numerous calculi in the right kidney of the patient. The largest stone was 9.5 mm (maximal diameter), while no stone presence was evidenced in the patient’s left kidney. The voiding cystourethrogram was normal, and the urine biochemistry investigation showed increased urine albumin (18 mg/dl) and microalbumin (103 mg/L) levels. Routine serum laboratory testing for urolithiasis was normal, failing to report any abnormal electrolyte count or any metabolic abnormality, such as the presence of hyperthyroidism. Moreover, arterial blood gas was normal (the bicarbonate’s count within the normal range), showing no acidosis or alkalosis. Finally, hypercalciuria was revealed after the 24 h urine examinations.

**Figure 1:**
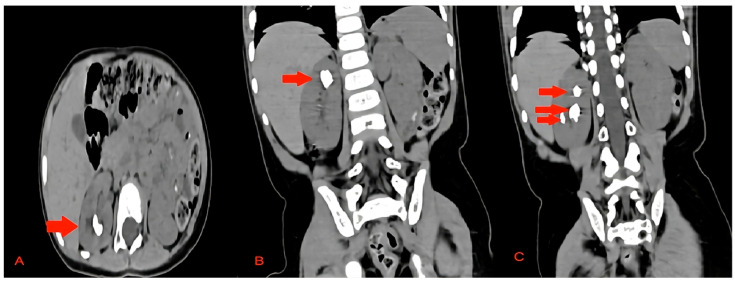
The preoperative CT scan of this case showing the stones in the right kidney. (**A**) Axial view of the upper pole stone, (**B**) coronal view of the upper pole stone, and (**C**) coronal view of the upper pole and middle pole stones. The stones are marked with red arrows.

**Figure 2:**
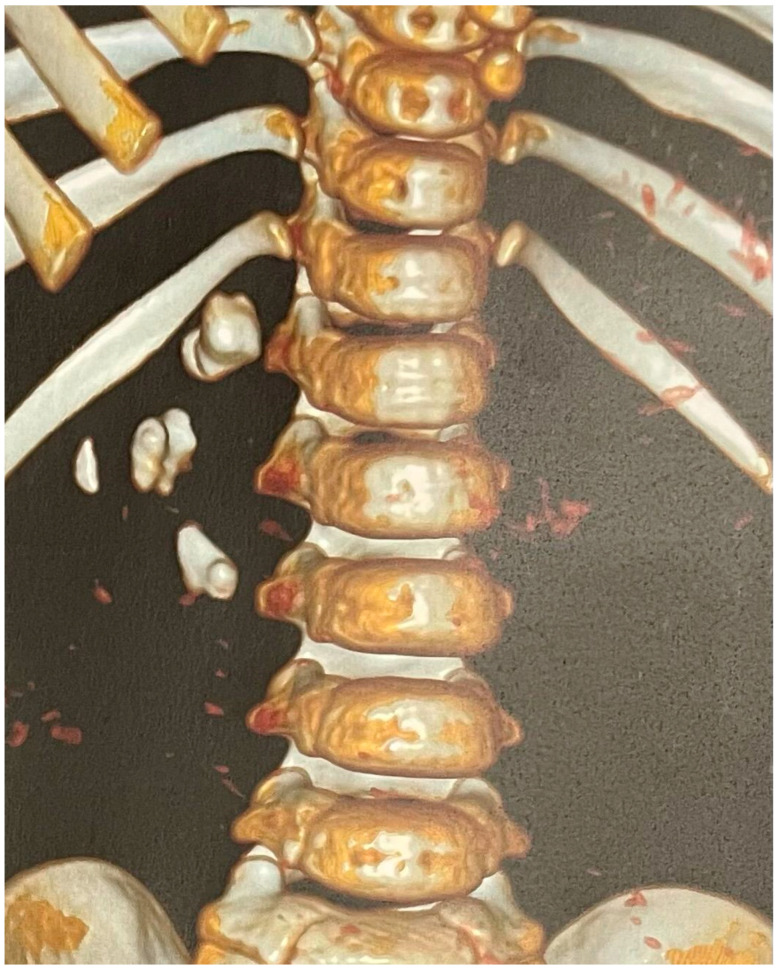
The 3D reconstruction of the preoperative CT scan of this case showing the stones in the right kidney.

A non-contrast low-dose kidney–ureters–bladder (KUB) computed tomography (CT) was performed, reporting six stones in the right kidney of the patient with a cumulative stone burden of 3.2 cm^2^ (two stones per calyx without filling the pelvis). The largest stone was located in the upper calyceal system, measuring 10 × 14 mm and 810 Hounsfield units (HUs) (Figure 1 and Figure 2).

**Figure 3:**
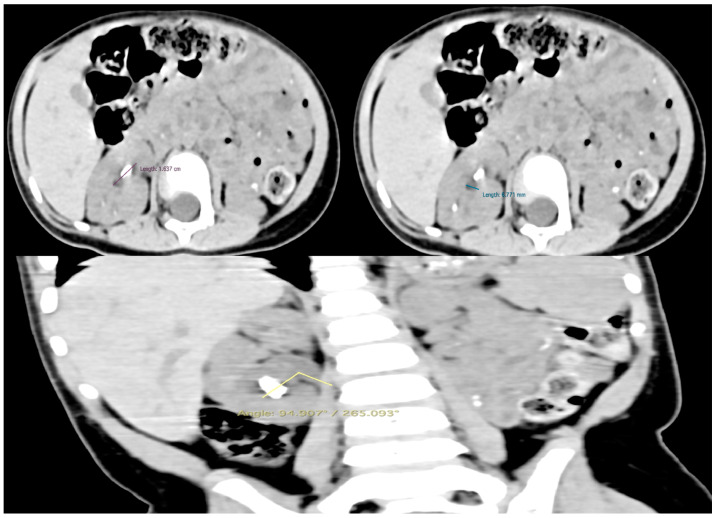
The size of the lower calyx and the infundibulopelvic angle. They were measured preoperatively to provide significant information for surgical planning decisions.

Two months later, a cystoscopy under general anesthesia was performed, followed by fluoroscopy of the right renal collecting system and the ureter. Fluoroscopy revealed the angle of the right ureteropelvic junction, while, after artificial dilation, the infundibulum of all the calyces seemed to be of adequate size for the maneuverability of the single-use flexible ureteroscope. Based on the preoperative non-contrast CT scan, the size of the lower calyx was 16.4 × 6.8 mm, and the infundibulopelvic angle was estimated to be 94.9°/265.1° (Figure 3). Consequently, based on the manufacturer characteristics of the single-use flexible ureteroscope utilized, the anatomy was convenient for the exploration of the whole calyx. It was then that the treatment plan was orientated towards fURSs. Finally, a 3.7 F/12 cm JJ stent was inserted.

### 2.2. Surgical Technique and Follow-Up

Two months after JJ placement, the patient underwent RIRS on the right kidney. At the time of surgery, the patient was 26 months old, and his weight was 10.3 kg, while a dose of ceftriaxone (based on a previous antibiogram) was administered intraoperatively. Under general anesthesia, the patient was placed in the lithotomy position.

**Figure 4:**
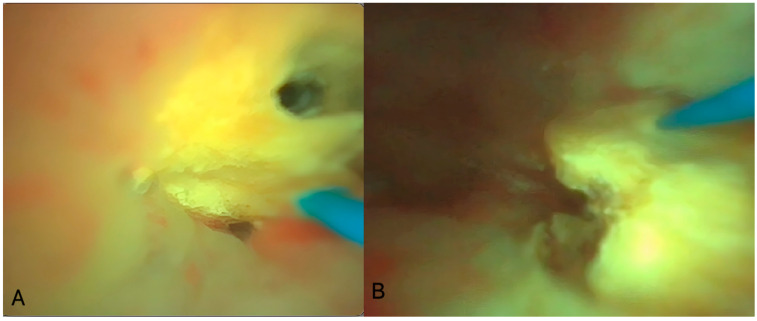
(**A**) Intraoperative image of laser lithotripsy during this case. (**B**) Another intraoperative image of endoscopic lithotripsy in this case.

Firstly, a cystoscopy was performed using a 9.5 F pediatric urethro-cystoscope (Karl Storz, Tuttlingen, Germany), and the JJ stent was removed. Afterward, a 0.018-inch diameter hydrophilic guidewire was placed on the right upper urinary tract, and a second safety 0.028-inch diameter hydrophilic-stiff guidewire was placed using a 10 F dual-lumen ureteral catheter (Cook Medical, Limerick, Ireland). Despite the normally small caliber of the pediatric ureter, it has the capability of extreme flexibility and dilatation if there is no stenotic part. The ureteral catheter was placed very gently without force. Thus, besides the second guidewire placement, it performed adequate dilatation for the semi-rigid ureteroscopy and the placement of the ureteral access sheath that followed. A semi-rigid ureteroscopy was performed using a 6/7.5 F ureteroscope (Karl Storz, Tuttlingen, Germany) to ensure that there were no stones in the ureter, followed by the placement of a 9.5/11.5 F ureteral access sheath (UAS) (Cook Medical, Limerick, Ireland) over a hydrophilic guidewire and under fluoroscopic guidance. The use of 9.5/11.5 F UAS was used to ensure decreased intrarenal pressure, considering that the size of single-use fURSs met the requirement of endoscope: the UAS ratio, as described by Fang et al. [8]. The UAS was placed just below the ureteropelvic junction, while contrast was injected to reveal the anatomy of the pelvicalyceal system and to define the exact position of the stones. An HP RIRS was performed using a 7.5 F single-use fURS (Pusen Medical Technology, Zhuhai, Guangdong, China) with the Ho/YAG laser device and a 272 μm fiber (Quanta, Quanta System, Samarate, Italy). Considering the large stone burden and the young age of the patient, fragmentation laser settings of 45 watts (either 1 J/45 Hz or 1.5 J/30 Hz) in the virtual basket pulse mode were used (Figure 4). 

**Figure 5:**
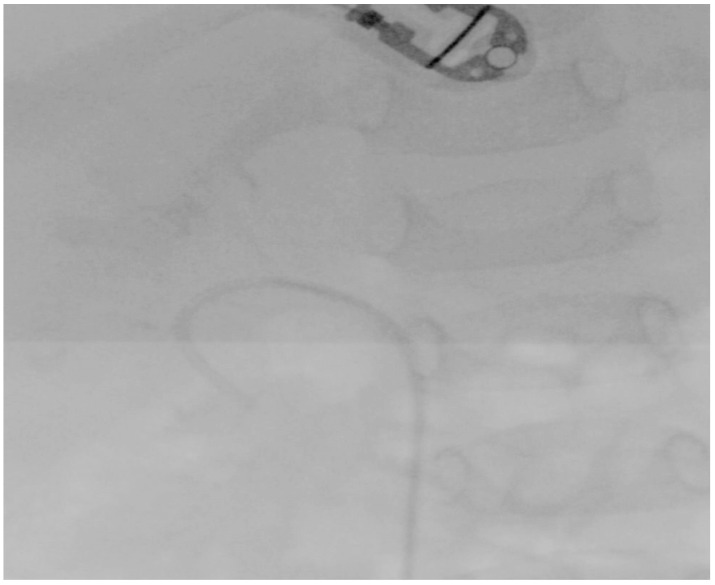
A fluoroscopy image after the end of the procedure showing no apparent stones.

All the stones were fragmented until a size of 2 mm was reached, which was considered the threshold for spontaneous passage [9]. The whole procedure was performed under irrigation of 40 mmHg automatic pressure and the application of a manual flush if needed. Finally, a 4.7 F/14 cm JJ stent (Cook Medical, Limerick, Ireland) and an 8 F Foley urethral catheter were placed after the completion of the lithotripsy (Figure 5).

The total operative and lithotripsy times were estimated at 90 and 75 min, respectively. Postoperatively, ceftriaxone was administered until discharge. The analgetic regimen included the use of paracetamol if needed. No complications (additional pain, hematuria, or fever) were recorded. Hemoglobin loss was calculated at 0.3 mg/dL, while the creatinine level decreased by 0.1 mg/dL. Additionally, the number of white blood cells and platelets on the first postoperative day was calculated at 9.97 million/μL and 338 million/μL, respectively. Moreover, postoperative sodium and potassium were 135 mmol/L and 4.1 mmol/L, respectively, while the SGOT and SGPT levels were normal (33 U/I and 16 U/I, respectively). The urethral catheter was removed on the first postoperative day, and the patient was discharged. JJ stent removal was scheduled one month postoperatively after the performance of ultrasound and kidney–ureter–bladder (KUB) X-ray, which did not detect >4 mm residual stone fragments. During the three-month follow-up, no complications occurred. Dust was macroscopically detected in the urine without the presence of small stone fragments. Three months postoperatively, the performed ultrasound and kidney-ureter-bladder (KUB) X-ray did not detect any significant residual fragments, while the patient was administered oral regimen for hypercalciuria by the pediatric nephrologist.

## 3. Discussion

In this study, a case report of a 26-month-old boy who underwent RIRS for multiple calyceal kidney stones with a cumulative stone burden of 3.2 cm^2^ is reported [10]. The patient underwent a JJ stent placement two months after the initial reference due to a refractory upper respiratory tract infection, while the RIRS was postponed for one additional month after the JJ placement due to the same cause. Despite the age of the patients and the large stone burden, the stone-free status seemed to be achieved in a single session, while HP lithotripsy seemed to be both a safe and effective treatment approach for this patient. The use of a single-use fURS was essential for the completion of the operation, as it provided the capability to approach all the calyces of the pelvicalyceal system and, consequently, to confront all the stones without the need for more interventional options, such as percutaneous nephrolithotripsy.

The incidence of pediatric urolithiasis varies among different regions. Turkey and Pakistan are the dominant countries associated with an endemic character of pediatric urolithiasis, while its incidence is continuously increasing in Western countries [3,11]. Calcium stones constitute the most common type of urinary stones in the pediatric population, affecting over 70% of patients. Uric acid and cystine stones are presented in 4–8% and 2–6% of cases, respectively. Additionally, in the pediatric population, struvite stones affect 5% of stone formers, mainly due to congenital anatomic anomalies [3]. Taking into consideration the high recurrence rate of pediatric urolithiasis, the risk factors should be investigated and treated in each patient [12]. There are many proven risk factors for pediatric urolithiasis, including age, obesity, diet, congenital anomalies, metabolic disorders, gender, diabetes, hypertension, and family history [13,14]. In recent years, the consumed water was also investigated for its potential contribution to the increased risk for stone formation. Based on the available literature, high magnesium and bicarbonate water may result in a reduction in the calcium oxalate crystals, while the struvite stone may be prevented by utilizing water with low calcium and magnesium levels [15]. On the other hand, Rossi et al. supported the use of oligomineral water, including high bicarbonate and low sulfate and magnesium levels, which may lead to a reduced rate of calcium oxalate crystals [16]. Further studies should be conducted for the precision of the optimal mineral composition for each stone type.

The integration of the single-use fURS constituted a new perspective in the management of kidney stones in adult patients. Despite not being superior regarding the surgical outcomes, the decreased cost per operation, the decreased risk of contamination, and the prevention of equipment damage constitute the advantages of the fURS [9]. Until now, many brands have created single-use fURSs of different sizes and characteristics. Nevertheless, their application in pediatric kidney stone management has been relatively delayed due to the considerations concerning the anatomy of the pediatric pelvicalyceal system, the safety and durability of the developing kidney tissues, and the total cost of the operation. Mille et al. compared the utilization of single-use and reusable fURSs in children with a median stone size of 13 mm. The authors supported the fact that single-use fURSs are not inferior to reusable ones, as they are associated with similar mean operation times (104.5 ± 73 min vs. 84 ± 15.9 min, *p* = 0.18), stone-free rates (88% vs. 84%, *p* = 0.39), and postoperative complication rates (4.6% vs. 5%, *p* = 0.81). Additionally, the total cost per operation was confirmed to be lower in the fURS group, while the difference did not reach a statistically significant level (EUR 1483.23 vs. EUR 798, *p* = 0.47) [9]. In our study, a single-use fURS was utilized, providing access to all the calyces and calyceal stones of the patient.

Despite the fact that the guidelines of the European Association of Urology propose percutaneous nephrolithotomy as the first-line treatment for kidney stones larger than 2 cm, RIRS may achieve acceptable outcomes in pediatric patients [3]. Zhang et al. compared mini-percutaneous nephrolithotomy (mPCNL) and RIRS in children with kidney stones larger than 2 cm. Based on their results, the median operative time was significantly diminished in the RIRS group by (25 (20–30) min versus 40 (25–65) min, *p* < 0.001), while the overall stone-free rates were similar (79.7% vs. 80.9%, *p* = 0.19). Nevertheless, the complication rate was significantly higher in the mPCNL group (27.4% vs. 52.5%, *p* = 0.01). After subgrouping based on the stone burden, in combination with the HU (cm^2^·HU), RIRS proved to be inferior in terms of the stone-free rate in the >5000 cm^2^·HU group (55.5% vs. 83.3%, *p* = 0.04), while the complication rate was similar (55.6% vs. 56.5%, *p* = 0.95) [17]. A randomized prospective study to compare mPCNL and RIRS was conducted by Mahmoud et al. The authors included 45 patients in each group, resulting in similar stone-free (88.9% vs. 95.6%, *p* = 0.238) and complication rates (6.7% vs. 15.6%, *p* = 0.401). Despite the similar mean operative times (88 ± 34.27 min vs. 89.11 ± 33.58 min, *p* = 0.877), the fluoroscopy and hospitalization times significantly favored the RIRS group (93.16 ± 35.15 s vs. 127.56 ± 54.97 s, *p* = 0.001 and 36.27 ± 11 h vs. 55.60 ± 37.75 h, *p* = 0.001, respectively). Nevertheless, mPCNL was associated with a significantly lower total mean cost (EUR 1210 ± 151.25 vs. EUR 733 ± 91.63, *p* < 0.001) [18]. Endoscopic combined intrarenal surgery (ECIRS) constitutes an alternative treatment approach in patients with multiple stones, including lower and upper pole stones. Li et al. presented the outcomes of ECIRS in 21 patients with multiple kidney stones. The stone-free rate was calculated at 85.7%, while the mean surgical time was 45 ± 12 min, compromising 31 ± 13 min of RIRS for the pelvic, upper poles, and middle pole stones and 14 ± 3 min of micro-PCNL for the lower pole stones. The Clavien–Dindo I and II complication rates were estimated at 23.8% and 4.8%, respectively [19]. In our study, RIRS was selected as the first-line treatment option, providing access to all the stones, while HP lithotripsy achieved adequate fragmentation based on intraoperative endoscopic and fluoroscopic views. Considering the promising outcomes of the RIRS in combination with novel HP lasers and the anatomy of the kidney (infundibulopelvic angles and infundibulum sizes), the surgical plan of our patient included the use of RIRS as a primary surgical approach and, in cases of intraoperative difficulties, the conversion to mPCNL. Finally, the operation was successfully completed without the need for conversion.

The lower pole in the stone position was considered a drawback of the RIRS due to the very steep angle and the difficulty of the fURS to approach it. The special anatomy of the pediatric pelvicalyceal system seemed to reinforce this drawback. Mosquera et al. conducted a study to investigate the safety and efficacy of RIRS in lower pole stones in pediatric patients. The authors revealed that the stone-free rates were 82.4% and 98.2% after the first and second operations, respectively. Moreover, the complication rate was low (7%), compromising only Clavien–Dindo I complications. Consequently, the authors supported the fact that RIRS may be considered as a first-line treatment for lower pole kidney stones [20]. In our case, the fURS provided adequate access to both lower pole stones, achieving acceptable fragmentation.

The introduction of multiple laser types in urology has led to the development of urological procedures. Ho/YAG and thulium fiber laser (TFL) seem to constitute the predominant laser in urological surgical practice. Candela et al. compared the use of the two aforementioned laser types in RIRS. The authors stated that the TFL was associated with a shorter mean operative time (64.3 min vs. 49.5 min, *p* = 0.024) and a lower complication rate (14.4% vs. 6.89%, *p* = 0.046). The stone-free rate was estimated to be similar in the two groups (81.4% vs. 89.7%, *p* = 0.45), while the univariate and multivariate analyses did not detect any correlation between the stone-free rate and the type of laser. However, pre-stenting was proved to be associated with improved stone-free rates [21]. In our study, the Ho/YAG laser was used with excellent lithotripsy outcomes, while pre-stenting seemed to be a crucial part of the success of the operation.

Over the last decade, the technological development of laser devices has led to the dominance of HP lithotripsy. Garcia-Rojo et al. compared the outcomes of HP and the low-power (LP) Ho/YAG laser in pediatric kidney urolithiasis. The authors mentioned that the HP group was associated with a significantly reduced mean operative time (64.29 min vs. 75.27 min, *p* = 0.018) and a better stone-free rate (81.4% and 59%, *p* < 0.001). The complication rates seemed to be similar, while the multivariate analysis indicated that LP, in combination with large stones, is associated with poor stone-free rates [22]. Madarriaga et al. conducted a study to investigate the effectiveness of HP lithotripsy in patients suffering from cystine kidney stones. The authors used a laser power of 56 watts (0.8 J/70 Hz), resulting in a stone-free rate of 59%, while the occurred complication rate was estimated at 18.2%. More precisely, intraoperatively, laser fiber rupture, intra-renal bleeding and a Grade II ureteral injury occurred without affecting the operation plans. Postoperatively, five Clavien–Dindo II and one Clavien–Dindo I complications were recorded [23]. The safety and efficacy of HP Ho/YAG lithotripsy were also evaluated by Bujons et al. in patients who underwent mPCNL for staghorn or complex kidney stones. The authors applied a median total power of 70 watts (range, 50–100 watts). The stone-free rate was estimated to be at 78%, while the hemoglobin drop was not significant (11.33 ± 1.20 mg/dL preoperatively vs. 10.55 ± 1.15 mg/dL postoperatively, *p* > 0.1). Two patients suffered from postoperative urinary tract infection (Clavien–Dindo II complication) [24]. In our study, a total power of 45 watts was utilized (either 1 J/45 Hz or 1.5 J/30 Hz) without the presence of intra- or postoperative complications, while the hemoglobin drop was minimal (0.3 mg/dL).

The age and the weight of patients constitute major considerations for the performance of operations in pediatric patients. Berrettini et al. investigated the safety and efficacy of RIRS with the use of UAS in pediatric patients under 20 kg. The authors included 16 patients with a mean weight of 14.88 ± 3.81 kg and a mean stone burden of 15.5 ± 3.8 mm. A 9.5/11.5 F UAS was placed in 93.8% of the patients, while a stone-free rate of 81.3% was initially achieved. The Clavien–Dindo II and III complication rates were recorded to be 12.5% and 6.3%, respectively [25]. The safety of RIRS in children under the age of 5 years was investigated by a multicenter retrospective study conducted by Faure et al. In total, 83 patients with a median age of 3.5 years and a median stone size of 13.5 mm were enrolled. The majority of the stones were located in the pelvic or lower calyx, while the UAS was successfully utilized in 91% of the operations. The stone-free rate was estimated to be 67.4% after the first procedure, while the total complication rate was calculated at 18.7%, including a case of forniceal rupture intraoperatively (Clavien–Dindo III complication) [26]. Despite being 26 months old and weighing 10.3 kg, our patient did not experience any operative or anesthesiology complications.

This study is associated with some limitations. First of all, it is a presentation of an individual case. However, there are restricted studies in the literature regarding the effectiveness and the outcomes of the use of single-use flexible ureteroscopy in toddlers with a stone burden over 2 cm^2^. Additionally, the operation was performed recently, and consequently, the family perspective and experience measurements are not available as the required follow-up period has not been completed. Further investigations should be conducted for the long-term outcomes of single-use flexible ureteroscopy in toddlers with a high stone burden.

## 4. Conclusions

The management of multiple or large kidney stones is very challenging in the pediatric population under the age of three years. The convenient preoperative planning and the appropriate use of the available equipment and its characteristics may lead to excellent outcomes accompanied by a reduced risk for complications.

## Data Availability

The data that support the findings of this study are available from the corresponding author, upon reasonable request.

## Abbreviation List

CT	computed tomography
ECIRS	endoscopic combined intrarenal surgery
fURS	flexible ureteroscope
HO/YAG	holmium/YAG
HP	high power
HU	Hounsfield units
KUB	kidney, ureter, and bladder
LP	low power
mPCNL	mini percutaneous nephrolithotripsy
RIRS	retrograde intrarenal surgery
TFL	thulium fiber laser
UAS	ureteral access sheath
